# Author Correction: Increasingly negative tropical water–interannual CO_2_ growth rate coupling

**DOI:** 10.1038/s41586-026-10147-w

**Published:** 2026-02-03

**Authors:** Laibao Liu, Philippe Ciais, Mengxi Wu, Ryan S. Padrón, Pierre Friedlingstein, Jonas Schwaab, Lukas Gudmundsson, Sonia I. Seneviratne

**Affiliations:** 1 Institute for Atmospheric and Climate Science, ETH Zurich, Zurich, Switzerland; 2 Laboratoire des Sciences du Climat et de l’Environnement, CEA-CNRS-UVSQ, Université Paris Saclay, Gif-sur-Yvette, France; 3 Joint Institute for Regional Earth System Science and Engineering (JIFRESSE), University of California, Los Angeles, Los Angeles, CA USA; 4 College of Engineering, Mathematics and Physical Sciences, University of Exeter, Exeter, UK

**Keywords:** Carbon cycle, Climate change

Correction to: *Nature* 10.1038/s41586-023-06056-x Published online 31 May 2023

In the version of the article initially published, the Wilcoxon signed-rank test was misapplied because bootstrapping artificially inflated the sample size for this test. Using a more suitable statistical method, the *P* values are slightly larger (<0.1 on average) than previously reported. The following two relevant corrections have been made: in the second paragraph of the “Observed climate–carbon coupling” section, the text “but water–CGR correlations are significantly different between the two periods (based on the Wilcoxon signed-rank test, *P* < 0.05) and become more negative over time (Fig. 1b and Extended Data Table 1)” has been replaced with “but water-CGR correlations significantly become more negative over time (*P* < 0.1 averaged over correlation metrics, Fig. 1b and Extended Data Table 1)”. Secondly, in Extended Data Table 1, the statistical test in the last column, descriptions in table headings and the caption have all been corrected. The original Extended Data Table 1 is available for comparison as Supplementary information accompanying this amendment. In addition, a typographical error in the first paragraph of the “Observed climate–carbon coupling” section has been corrected to “Mount Agung (1963 and 1964)”. Our conclusions remain robust when evaluated with appropriate statistical tests, supported by the consistency across all the metrics and multiple lines of evidence in the paper. We thank Dr D. Kennedy, A. Trugman, D. Lawrence, S. Swenson, and I. Simpson for bringing the issue to our attention.

**Supplementary information** is available in the online version of this amendment.

## Supplementary information

Original, uncorrected Extended Data Table 1

