# Supplementary material for: Increasingly negative tropical water–interannual CO2 growth rate coupling
Source: Nature. 2023 May 31;618(7966):755–60. doi: 10.1038/s41586-023-06056-x (PMC10284699; doi:10.1038/s41586-023-06056-x)
Supplement: Supplementary file 1 — Supplementary Information Supplementary Figs. 1–15 and Table 1. [file 41586_2023_6056_MOESM1_ESM.pdf]

nature portfolio

https://doi.org/10.1038/s41586-023-06056-x

Supplementary information

Increasingly negative tropical water–interannual CO_2 growth rate coupling

In the format provided by the authors and unedited

Supplementary Information for the manuscript

"Increasingly negative tropical water - interannual CO_2 growth rate coupling"

Authors:

Laibao Liu[1]*, Philippe Ciais[2], Mengxi Wu[3], Ryan S. Padrón[1], Pierre Friedlingstein[4], Jonas Schwaab[1], Lukas Gudmundsson[1], and Sonia I. Seneviratne[1]

Affiliations:

[1] Institute for Atmospheric and Climate Science, ETH Zurich, Zurich, Switzerland

[2] Laboratoire des Sciences du Climat et de l'Environnement, CEA/CNRS/UVSQ/Université Paris Saclay, Gif-sur-Yvette, France

[3] Joint Institute for Regional Earth System Science and Engineering (JIFRESSE), University of California, Los Angeles

[4] College of Engineering, Mathematics and Physical Sciences, University of Exeter, Exeter EX4 4QE, UK

*Corresponding author: laibao.liu@env.ethz.ch

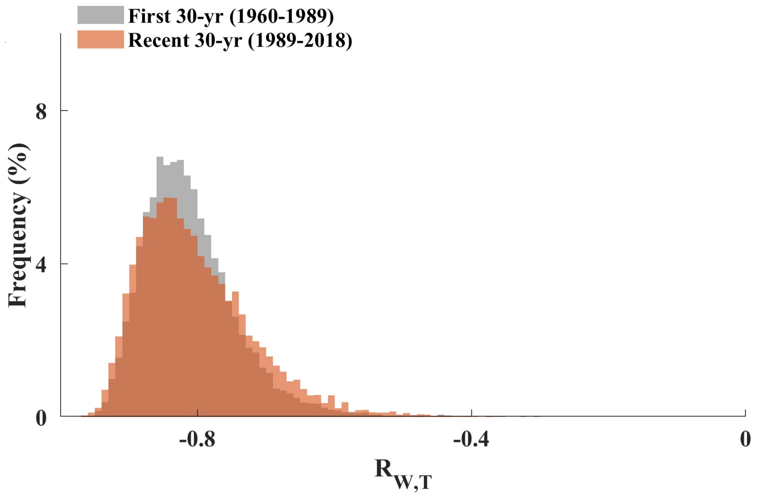

Supplementary Figure 1. Tropical land water-temperature interannual correlations. Histograms of tropical water-temperature interannual correlations in the first three decades (1960-1989) and in the recent three decades (1989-2018), derived using 5000 bootstrapping repeats. Both tropical WS and LagP are used to represent tropical water availability (W). R(W,T) refers to the combination of R(WS,T) and R(LagP,T).

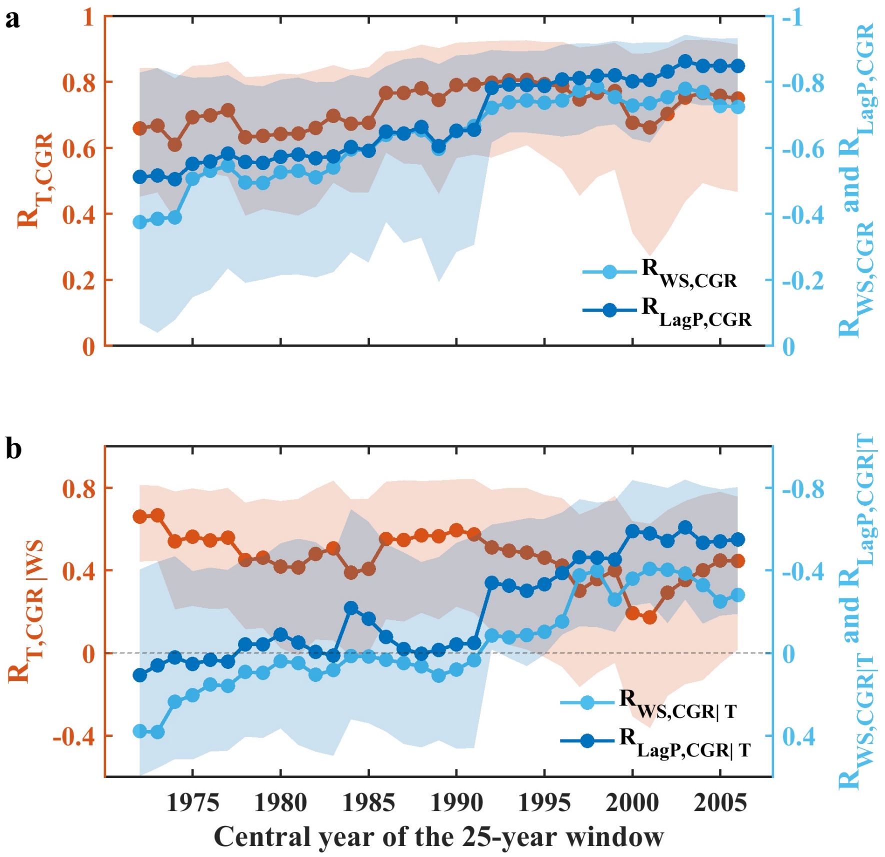

Supplementary Figure 2. Tropical land climate-carbon interannual correlations based on a 25-year moving window. **a,** Changes in interannual correlations of CGR with tropical temperature and tropical water over time by applying a 25-year moving time window. Both tropical WS and LagP are used to represent tropical water availability. Shaded areas represent the 95% confidence interval, derived using 5000 bootstrapping repeats. For a better readability, only the 95% confidence interval of R(T,CGR) and R(LagP,CGR) are plotted. In each window, all variables are detrended at yearly time scale, the central year is labeled on the horizontal axis (for instance, 1975 represents the period of 1963-1987). **b,** Same as **a**, but showing the partial correlations of CGR to tropical temperature and tropical water after controlling tropical water and tropical temperature, respectively. For a better readability, only the 95% confidence interval of R(T,CGR|WS) and R(LagP,CGR|T) are plotted.

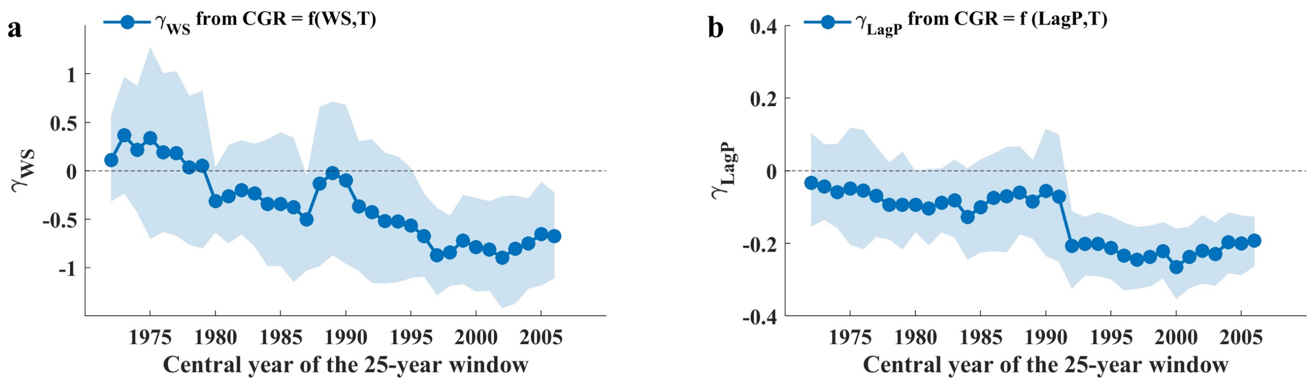

Supplementary Figure 3. Interannual sensitivity of CGR to tropical water based on a 25-year moving window. **a,** CGR is the function of tropical temperature and tropical WS. Sensitivity of CGR to tropical WS variation is estimated from the Ridge regression. Each dot indicates a 25-year period. The central year of the time window is labeled on the horizontal axis. Shaded areas represent the 95% confidence interval, derived using 5000 bootstrapping repeats. **b,** same as a, but replacing tropical WS with tropical LagP.

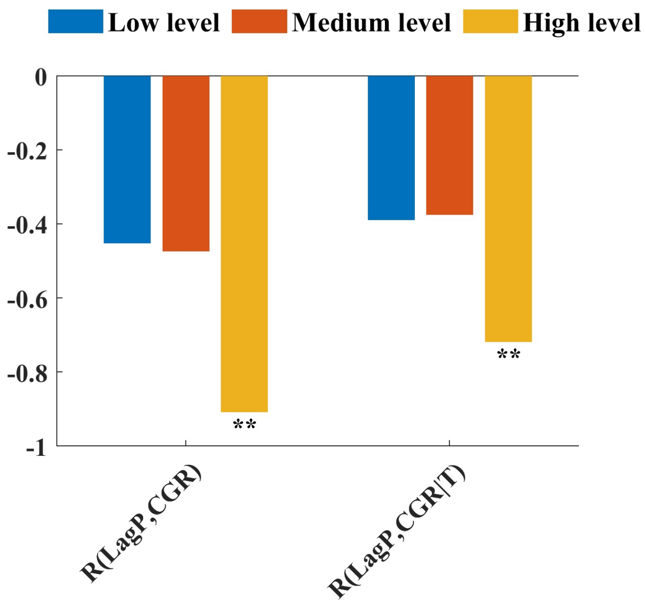

Supplementary Figure 4. Dependence of tropical water-CGR correlations on spatial coherence during 1960-2018.

Same as Fig. 2c, but replacing tropical WS with tropical LagP.

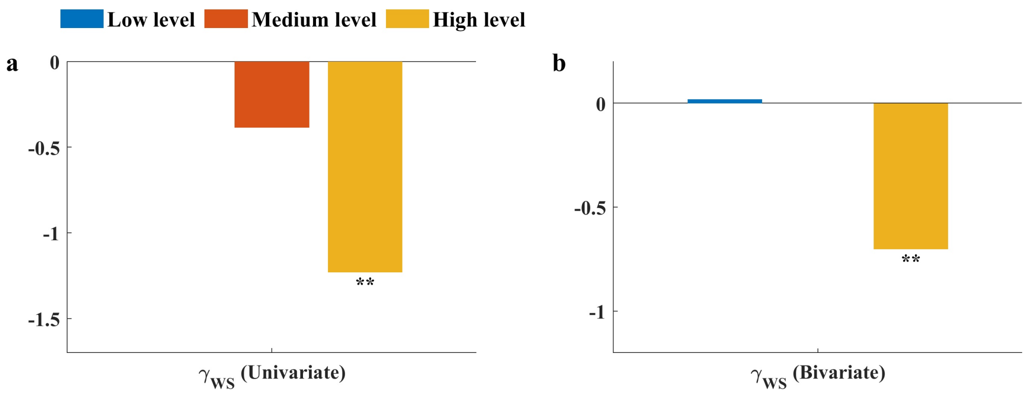

Supplementary Figure 5. Dependence of tropical water-CGR sensitivity on spatial coherence during 1960-2018. Years in each group of spatial coherence is the same as that in Fig. 2a. Univariate and bivariate sensitivity is estimated using the OLS regression and Ridge regression, respectively. The unit of this sensitivity is PgC year^{-1} per Tt H_2O. The best estimate of sensitivity of CGR to tropical WS is shown. ** indicates a significant sensitivity at $P<0.05$.

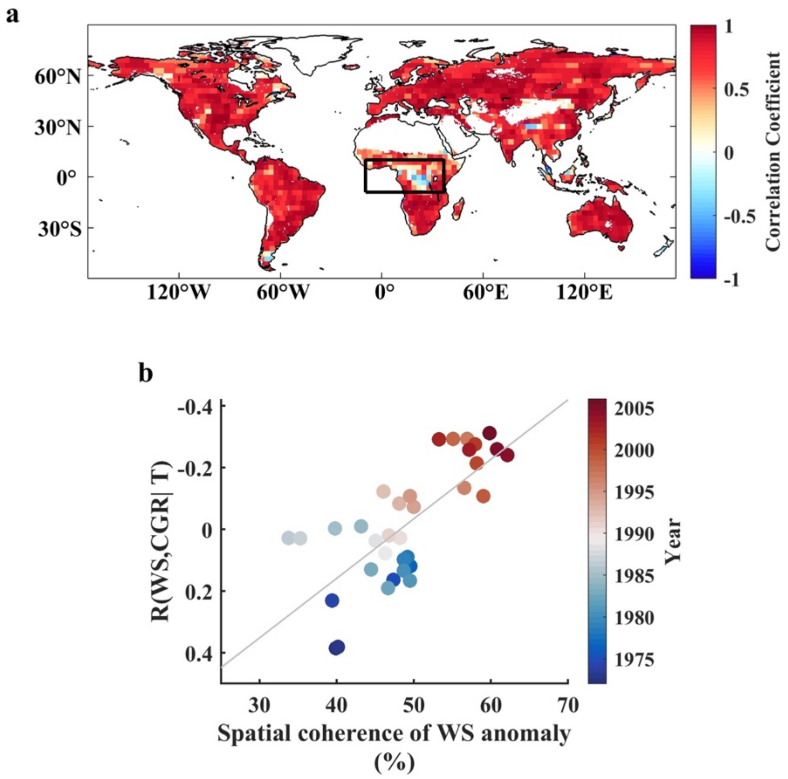

Supplementary Figure 6. Robustness of the dependence of tropical water-CGR correlation on spatial coherence. a, Spatial distribution of the interannual correlation of original WS to reconstructed WS at the yearly scale. A black rectangle indicates the areas of central Africa to be excluded from this analysis, covering 10°W to 38° E, 9°S to 10° N; **b,** Partial correlation between tropical WS and CGR after controlling tropical temperature versus the degree of spatial coherence in tropical WS anomaly. Each dot indicates a 25-yr moving window. The central year of the moving time window is indicated by the color.

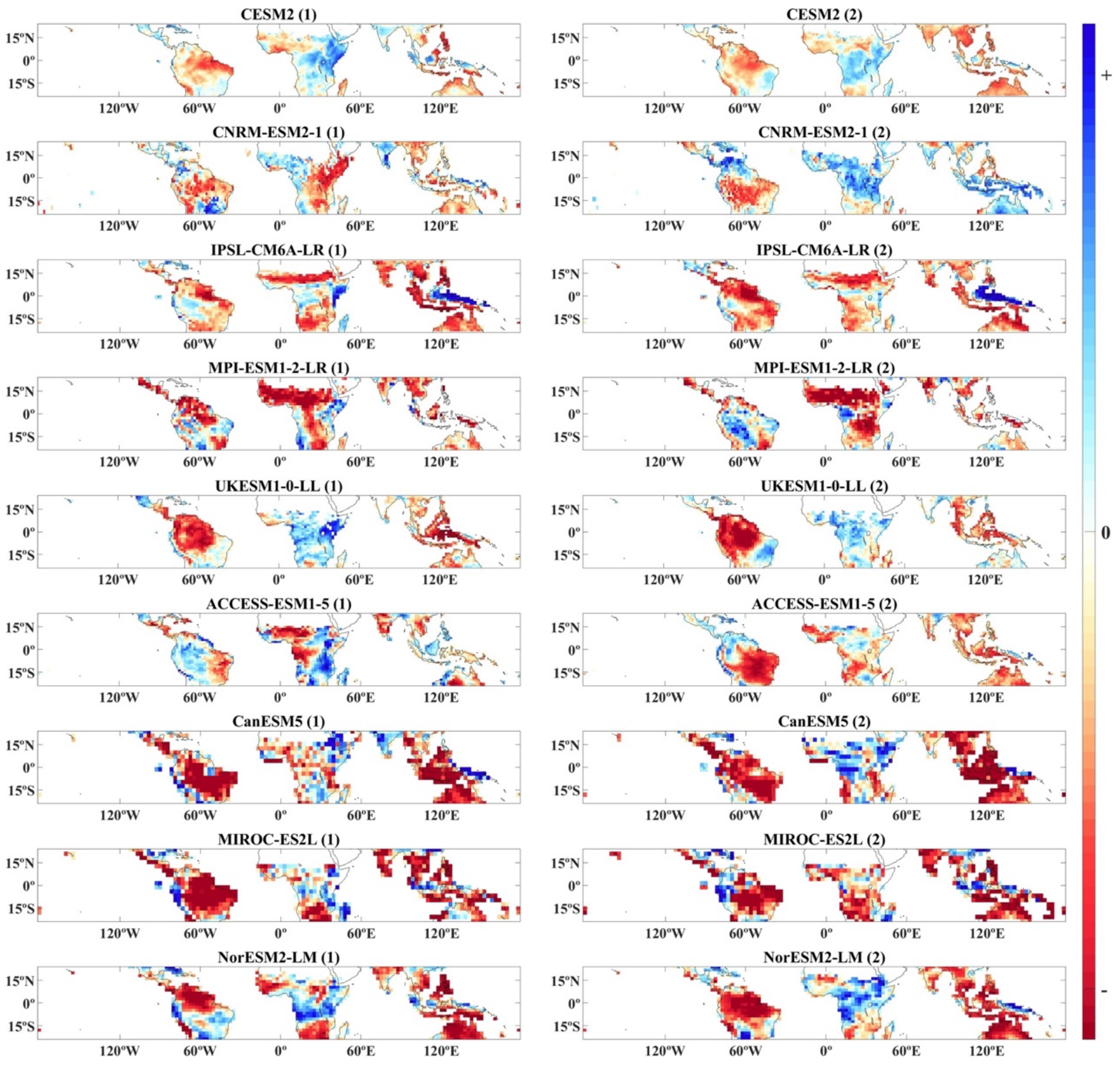

Supplementary Figure 7. Spatial patterns of the leading EOF of yearly tropical soil moisture anomalies in CMIP6 coupled models. The bracket (1) and bracket (2) indicate the time period of 1960-1986 and 1988-2014, respectively. Regions with sparse vegetation are excluded. Colors indicate the sign of values; magnitudes of values are not informative and are omitted for clarity. For each model (from top to bottom), the total spatial variance explained by the leading EOF are: 13.3% (1), 22.0% (2); 11.4% (1), 12.3% (2); 18.8% (1), 17.8% (2); 18.6% (1), 16.8% (2); 14.7% (1), 13.3% (2); 12.6% (1), 17.7% (2); 13.4% (1), 19.6% (2); 18.4% (1), 19.0% (2); 16.3% (1), 17.0% (2).

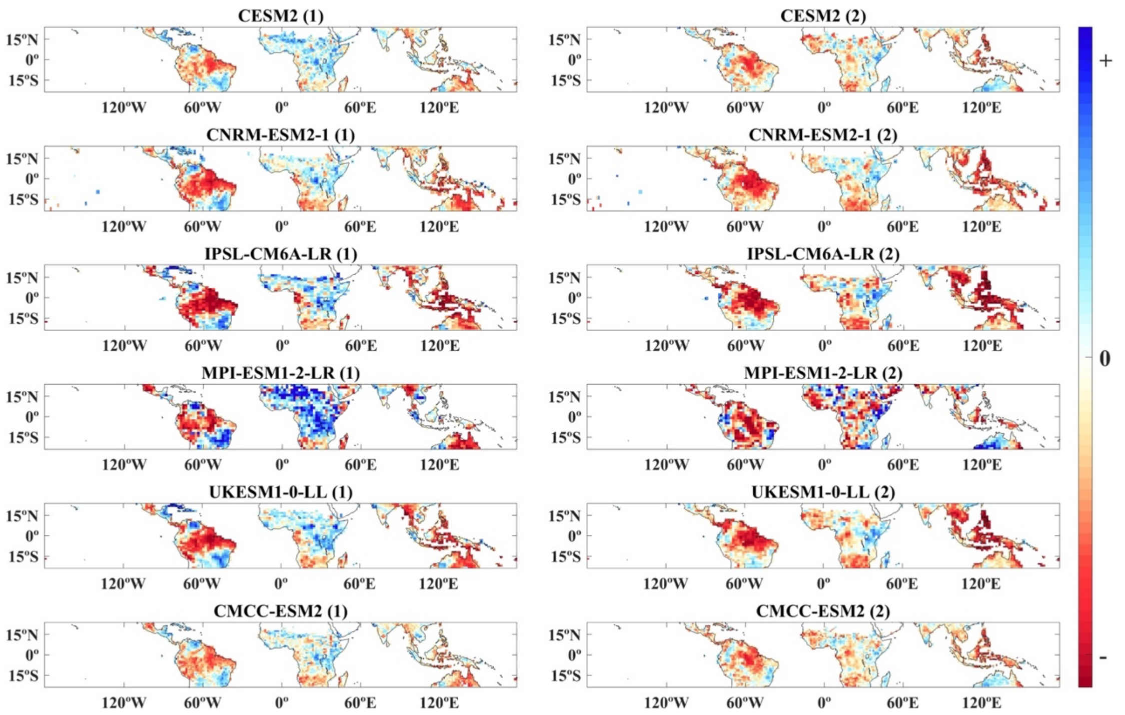

Supplementary Figure 8. Spatial patterns of the leading EOF of yearly tropical soil moisture anomalies in CMIP6 offline models. The bracket (1) and bracket (2) indicate the time period of 1960-1986 and 1988-2014, respectively. Regions with sparse vegetation are excluded. Colors indicate the sign of values; magnitudes of values are not informative and are omitted for clarity. For each model (from top to bottom), the total spatial variance explained by the leading EOF are: 12.6% (1), 12.5% (2); 12.7% (1), 13.7% (2); 12.7% (1), 14.3% (2); 15.9% (1), 13.7% (2); 13.7% (1), 14.5% (2); 12.2% (1), 12.4% (2).

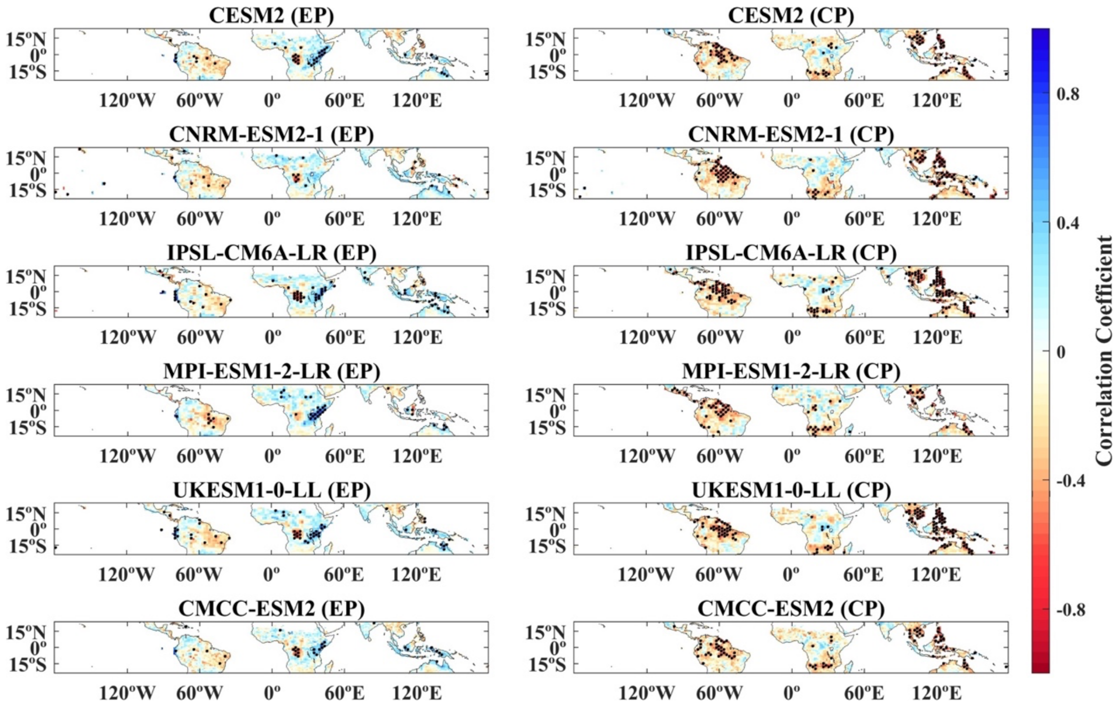

Supplementary Figure 9. Spatial distribution of the interannual correlations between simulated soil moisture anomaly and ENSO in CMIP6 offline models. The bracket (EP) and bracket (CP) indicate EP ENSO and CP ENSO during 1989-2018, respectively. Regions with significant correlation ($P<0.05$) are stippled.

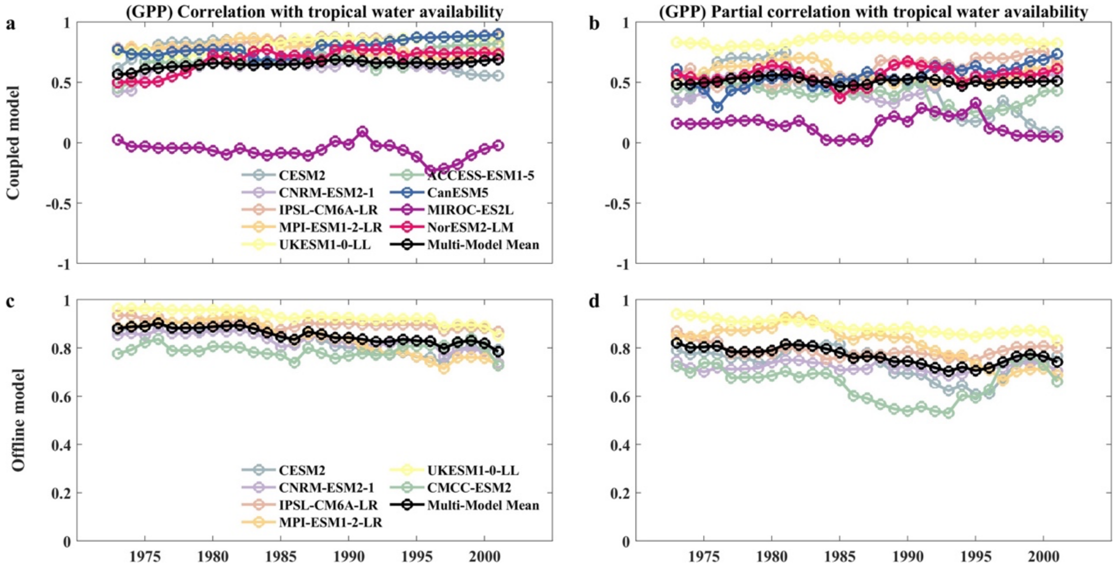

Supplementary Figure 10. Interannual correlations and partial correlations between gross primary production (GPP) and tropical water availability. Years labeled on the horizontal axis indicate the central year of the 27-year moving time window (all variables detrended at yearly scale in each corresponding window). Models are based on tropical total soil moisture and simulated global net ecosystem exchange.

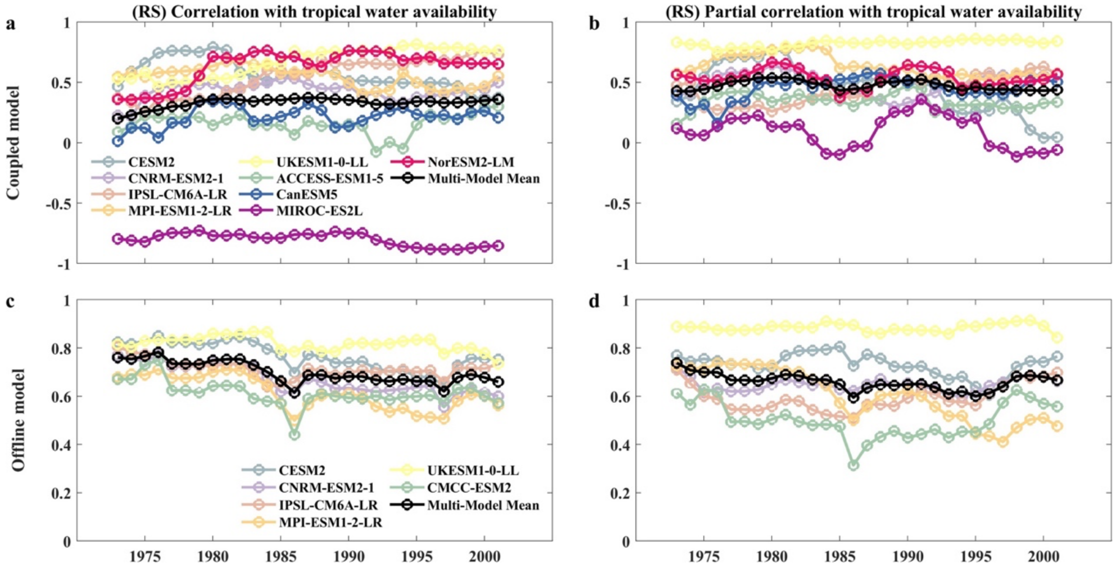

Supplementary Figure 11. Interannual correlations and partial correlations between ecosystem respiration (RS) and tropical water availability. Years labeled on the horizontal axis indicate the central year of the 27-year moving time window (all variables detrended at yearly scale in each corresponding window). Models are based on tropical total soil moisture and simulated global net ecosystem exchange.

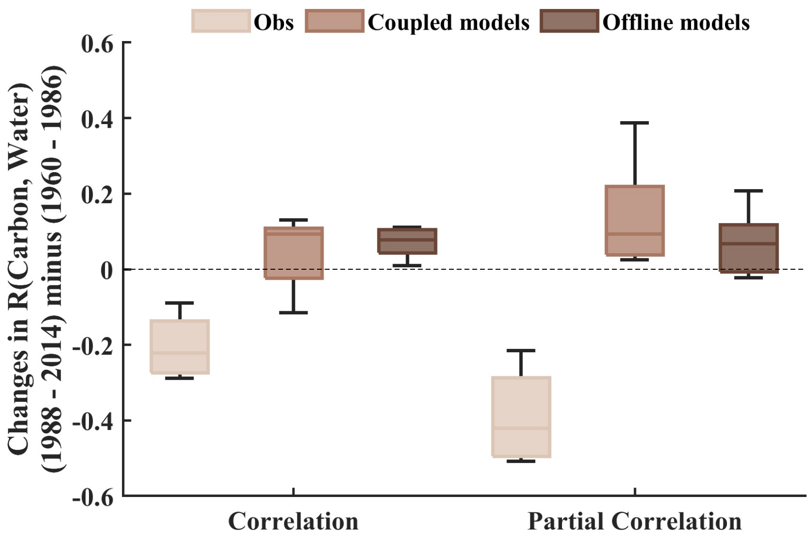

Supplementary Figure 12. Same as Figure 3a, but replacing NEE with net biome production.

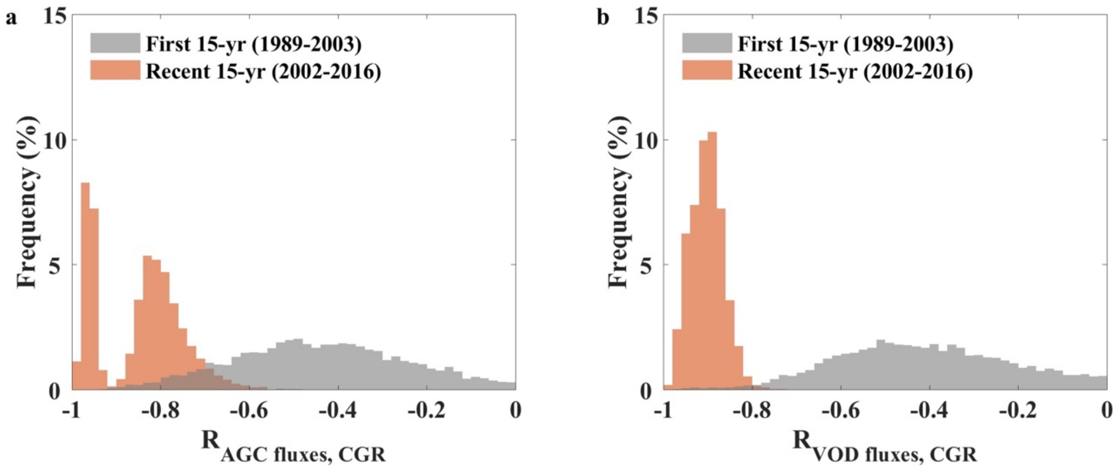

Supplementary Figure 13. Interannual correlations of CGR to semiarid VOD fluxes and VOD-derived AGC fluxes. **a,** Histograms of semiarid AGC-CGR correlations in the first 15-yr (1989-2003) and in the recent 15-yr (2002-2016), derived using 5000 bootstrapping repeats. The tropical semiarid annual AGC fluxes were calculated using net AGC changes for individual years (compared to the previous year). **b,** same as **a**, but replacing with semiarid VOD-AGC with semiarid VOD.

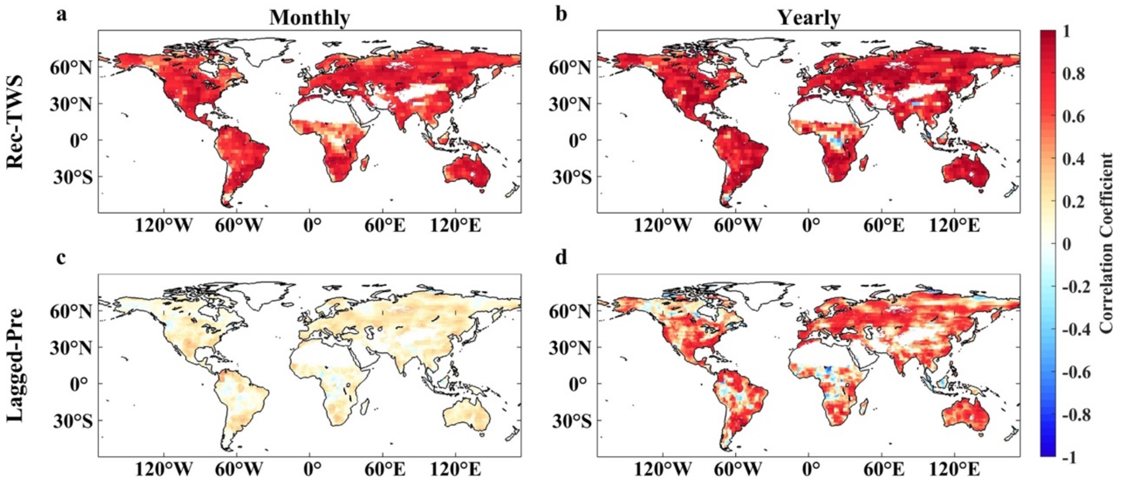

Supplementary Figure 14. Spatial distribution of the interannual correlation of original terrestrial water storage to reconstructed terrestrial water storage and 6-month lagged precipitation at monthly and yearly scale during 2002-2016.

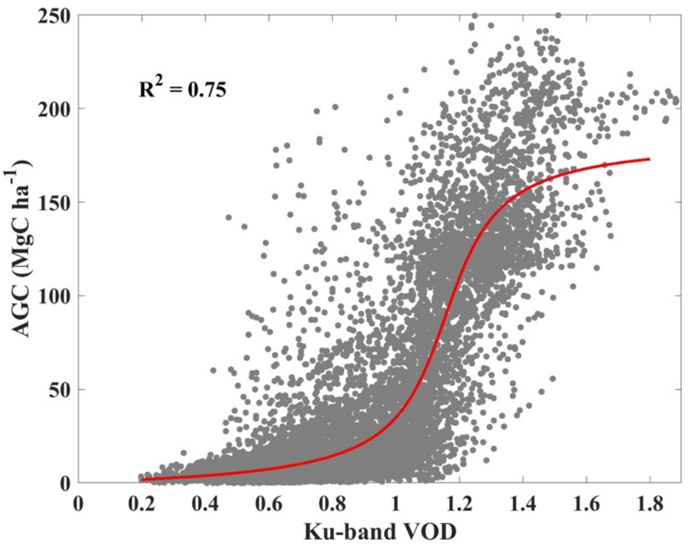

Supplementary Figure 15. Relationship between Ku-VOD and aboveground carbon density in tropics. The spatial scatter plot between yearly mean 0.5° Ku-VOD values and AGC over the tropical regions. Each point indicates one grid cell. Red line shows the fitted relationships. The values of fitted parameter in the empirical function are as follows: a = 184.3; b = 7.978; c=1.156; d= 0.3861.

Supplementary Table 1. Interannual correlation of original terrestrial water storage to reconstructed terrestrial water storage and 6-month lagged precipitation at regional scale during 2002-2016. **Significance correlation at P<0.05.

**Interannual correlation to original terrestrial water storage**	**Reconstruction**	**Lagged Precipitation**
	**yearly**	**yearly**
Tropical	0.96**	0.89**
Tropical forest	0.91**	0.74**
Tropical semiarid	0.98**	0.92**
