# Supplementary material for: Author Correction: Increasingly negative tropical water–interannual CO2 growth rate coupling
Source: Nature. 2026 Feb 3;650(8102):E12. doi: 10.1038/s41586-026-10147-w (PMC12916495; doi:10.1038/s41586-026-10147-w)
Supplement: Supplementary file 1 — Original, uncorrected Extended Data Table 1 [file 41586_2026_10147_MOESM1_ESM.pdf]

nature portfolio

https://doi.org/10.1038/s41586-026-10147-w

Supplementary information

Author Correction: Increasingly negative tropical water–interannual CO_2 growth rate coupling

In the format provided by the authors and unedited

Original, uncorrected Extended Data Table 1

Extended Data Table 1 | Interannual correlation and sensitivity of CGR to tropical water

Metric	Method	Proxy of Water	Metrics during first 30-year	Probability that Metrics during first 30-year is different from 0	Metrics during recent 30-year	Probability that Metrics during recent 30-year is different from 0	Test whether the Means of Metrics during the two 30-year periods is equal (Wilcoxon signed-rank test)
Correlation	R(W,CGR)	WS	-0.53 ± 0.14**	99.9%	-0.71 ± 0.1**	99.9%	Not, P<0.01
		LagP	-0.58 ± 0.15**	99.2%	-0.80 ± 0.06**	99.9%	Not, P<0.01
	R(W,CGR|T)	WS	0.13 ± 0.21	44.5%	-0.36 ± 0.20*	90.4%	Not, P<0.01
		LagP	-0.04 ± 0.24	2.7%	-0.56 ± 0.14**	99.6%	Not, P<0.01
Sensitivity	Univariate (OLS)	WS	-0.95 ± 0.27**	99.9%	-1.26 ± 0.23**	99.9%	Not, P<0.01
		LagP	-0.24 ± 0.07**	99.4%	-0.33 ± 0.05**	99.9%	Not, P<0.01
	Bivariate (Ridge)	WS	-0.05 ± 0.33	15.6%	-0.66 ± 0.26**	98.9%	Not, P<0.01
		LagP	-0.06 ± 0.1	43.4%	-0.22 ± 0.05**	99.9%	Not, P<0.01
	Bivariate (OLS)	WS	0.33 ± 0.55	43.4%	-0.68 ± 0.44*	90.4%	Not, P<0.01
		LagP	-0.03 ± 0.13	4.9%	-0.24 ± 0.07**	99.7%	Not, P<0.01
				55.3% (On average)		97.9% (On average)	

Estimates are derived from 5000 bootstrapping repeats by randomly selecting years without volcano perturbations in each sub-period. The mean and one standard deviation are presented for the metric. The probability that the metric is different from 0 and the corresponding P value are computed by inverting the corresponding confidence intervals. For instance, the probability that the metric is different from 0 is 96% suggests that the 96% confidence level of the metric does not include 0 and the corresponding P value is 0.04. ** and * indicate a significant sensitivity or correlation at $P < 0.05$ and $P < 0.1$, respectively.
